# ECE-CYC1 Transcription Factor CmCYC1a May Interact with CmCYC2 in Regulating Flower Symmetry and Stamen Development in *Chrysanthemum morifolium*

**DOI:** 10.3390/genes16020152

**Published:** 2025-01-26

**Authors:** Yi Yang, Ming Sun, Cunquan Yuan, Qixiang Zhang

**Affiliations:** 1 School of Architecture and Urban Planning, Anhui Jianzhu University, Hefei 230601, China; 2 Beijing Key Laboratory of Ornamental Plants Germplasm Innovation & Molecular Breeding, National Engineering Research Center for Floriculture, Beijing Laboratory of Urban and Rural Ecological Environment, Engineering Research Center of Landscape Environment of Ministry of Education, Key Laboratory of Genetics and Breeding in Forest Trees and Ornamental Plants of Ministry of Education, School of Landscape Architecture, Beijing Forestry University, Beijing 100083, China

**Keywords:** *Chrysanthemum morifolium*, capitulum, ECE, CYC1, flower symmetry, stamen development

## Abstract

Background: The attractive inflorescence of *Chrysanthemum morifolium*, its capitulum, is always composed of ray (female, zygomorphy) and disc (bisexual, actinomorphy) florets, but the formation mechanism remains elusive. The gene diversification pattern of the ECE (CYC/TB1) clade has been speculated to correlate with the capitulum. Within the three subclades of ECE, the involvement of CYC2 in defining floret identity and regulating flower symmetry has been demonstrated in many species of Asteraceae, including *C. morifolium*. Differential expression of the other two subclade genes, CYC1 and CYC3, in different florets has been reported in other Asteraceae groups, yet their functions in flower development have not been investigated. Methods: Here, a CYC1 gene, *CmCYC1a*, was isolated and its expression pattern was studied in *C. morifolium*. The function of *CmCYC1a* was identified with gene transformation in *Arabidopsis thaliana* and yeast two-hybrid (Y2H) assays were performed to explore the interaction between CmCYC1 and CmCYC2. Results: *CmCYC1a* was expressed at higher levels in disc florets than in ray florets and the expression of *CmCYC1a* was increased in both florets during the flowering process. Overexpression of *CmCYC1a* in *A. thaliana* changed flower symmetry from actinomorphic to zygomorphic, with fewer stamens. Furthermore, CmCYC1a could interact with CmCYC2b, CmCYC2d, and CmCYC2f in Y2H assays. Conclusions: The results provide evidence for the involvement of *CmCYC1a* in regulating flower symmetry and stamen development in *C. morifolium* and deepen our comprehension of the contributions of ECE genes in capitulum formation.

## 1. Introduction

The capitulum, a distinctive head-like inflorescence, is considered a pivotal morphological innovation for the evolutionary success of Asteraceae [1]. The inflorescence is composed of multiple florets on a single receptacle and surrounded by involucral bracts. These florets are usually divided into ray and disc florets, which are monosymmetric female (or sterile) and polysymmetric bisexual flowers, respectively [2]. The sophisticated and fascinating inflorescence architecture has attracted the attention of many botanists.

The ECE clade, known as the CYCLOIDEA (CYC)/TEOSINTE BRANCHED 1 (TB1) clade of the TCP family, primarily participates in the formation of flowers or branches from axillary meristems [3]. Phylogenetic analysis suggests that the ECE clade has experienced two rounds of gene duplication within core eudicots, resulting in three subclades: CYC1, CYC2, and CYC3 [4]. Among them, the key role of the CYC2 subclade in regulating flower symmetry has been a hot issue since the *CYC* gene was first characterized in *Antirrhinum majus* [5]. *CYC* and its paralog *DICHOTOM (DICH)* are continually expressed in dorsal regions of floral meristems and dorsal primordia at later stages, affecting flower symmetry, petal size, and petal shape and forming a staminode [6,7]. In *Helianthus*, *Gerbera*, *Senecio*, *Chrysanthemum*, and other Asteraceae groups, CYC2 genes are mainly expressed in ray florets, with low or no expression in disc florets. Functional analyses also indicated their independent recruitment in differentiating floret identity [8,9,10,11,12,13,14,15].

*Arabidopsis TCP18/BRANCHED 1 (BRC1)* is a CYC1 gene closely related to maize *TB1*, which suppresses axillary organ growth and generates female inflorescences [16]. In *Arabidopsis thaliana*, BRC1 promotes the growth arrest of axillary buds and interacts with FT and TSF proteins to prevent floral transition [17]. Unfortunately, although *BRC1* and *TCP12/BRC2*, CYC3 genes, are both expressed in the provascular tissue of flowering buds, their functions during flower development remain unclear [18]. In *Petunia axillaris*, three CYC1 genes (*PaTCP18a/b/c*) are expressed in inflorescences and flower buds, and they are differentially expressed in the petals of two selfing lines with small and large flowers, suggesting a possible role in flower development and petal growth [19]. However, up to now, information on the function of CYC1 genes in floral development has been quite limited.

Throughout the angiosperms, ECE genes have experienced multiple independent and lineage-specific duplications [20]. The duplication of CYC1 genes may provide the basis for the change in flowers from solitary to complicated inflorescences (including the capitulum) in Campanuloideae [21]. It is also hypothesized that the two plant groups with a capitulum, Dipsacaceae and Asteraceae, may have taken advantage of ECE gene duplication [2,22,23]. In Dipsacaceae, radiate species bearing both ray and disc florets have more than two times the number of ECE genes than discoid species, which contain only disc florets [23]. In *Anacyclus clavatus* [24], *C. morifolium* [25], *Helianthus annuus* [8], and *Gerbera hybrida* [11] (Asteraceae), ECE genes have also undergone several rounds of gene duplication, all resulting in 10 ECE members. Furthermore, genome-wide analyses have identified 13 ECE members in *C. lavandulifolium* [26]. *GhCYC10* and *HaCYC1a*, two CYC1 genes, are highly expressed in ovary tissue, as are CYC2 genes. Notably, in contrast to CYC2 genes, *HaCYC1a* and *ClCYC1a* are expressed at higher levels in disc florets than in ray florets [11]. In *Knautia macedonica* (Dipsacaceae), *KmCYC1* is significantly differentially expressed in the ventral, lateral, and dorsal petals of external florets and in the ventral petals of external and internal florets, as is *KmCYC2*, indicating a potential role in determining flower symmetry [27]. Moreover, yeast two-hybrid (Y2H) assays performed in gerbera (*G. hybrida*) and sunflower (*H. annuus*) have identified interactions between the proteins belonging to the three different subclades of ECE [11]. Therefore, CYC1, CYC2, and CYC3 subclades may be functionally related, and CYC1 and CYC3 genes may also participate in the formation of a head-like inflorescence.

*C. morifolium*, a typical example used to explore flower symmetry in Asteraceae, has a rich variety of flower types and has been widely planted around the world. However, the detailed genetic mechanism regulating the complex chrysanthemum inflorescence still remains unknown [28]. Our previous studies have identified six CYC2 genes and confirmed the interactions between CYC2 transcription factors in regulating flower symmetry [14,29]. We wondered if CYC1 genes play any role in chrysanthemum flower development and whether they are in any way related to CYC2 genes. RNA-Seq analysis of the chrysanthemum cultivar ‘Jinba’ has identified two CYC1 genes and they were highly expressed in immature inflorescences [25]. Chen et al. have reported a CYC1 gene, *DgBRC1*, from ‘Jinba’ and found it to be functionally conserved in regulating lateral branching [30]. However, the function of *DgBRC1* in flower development was not mentioned. In this study, we isolated another CYC1 gene, *CmCYC1a*, and explored its expression patterns during inflorescence development. Overexpression of *CmCYC1a* in *A. thaliana* significantly altered flower symmetry and the number of stamens. Additionally, Y2H assays were performed to investigate pairwise protein–protein interactions between CmCYC1a and CmCYC2. Our results provide a candidate gene, *CmCYC1a*, for regulating flower symmetry and stamen development which may act together with CmCYC2 in the formation of the chrysanthemum capitulum. This study is an essential step toward deciphering the involvement of three subclades of ECE genes in determining the morphogenesis of the capitulum.

## 2. Materials and Methods

### 2.1. Plant Material

‘Fen Ditan’, a cultivar of chrysanthemum with two whorls of ray florets and several whorls of disc florets, was obtained by hybridization; it has been used as one of the models in the study of chrysanthemum floral development, particularly concerncing flower symmetry [31,32,33,34,35,36]. Here, ‘Fen Ditan’ was grown under a photoperiod of 8 h of light (24 °C) and 16 h of darkness (20 °C), while *A. thaliana* (Columbia) and tobacco (*Nicotiana benthamiana)* were planted under the opposite photoperiod condition at 22 °C/19 °C and 27 °C/21 °C, respectively.

### 2.2. Gene Isolation

Total RNA was extracted from chrysanthemum inflorescences with a Plant RNA Kit (Omega, Norcross, GA, USA), followed by measurement of its quality and quantity on a spectrophotometer (Thermo Fisher, Waltham, MA, USA). cDNA synthesis was then performed with a One-Step gDNA Removal kit and cDNA Synthesis SuperMix (Transgen, Beijing, China). *CmCYC1a*-F1/R1 primers (Table S1) were used to amplify the coding sequence (CDS) of *CmCYC1a* (MK628725). Primers for the cloning of six *CmCYC2* genes, *CmCYC2a*-*CmCYC2f* (KU595426-KU595431), were reported before [14].

### 2.3. Phylogenetic Analysis

Sequences of ECE proteins from *A. majus*, *A. thaliana*, *C. morifolium*, *G. hybrida*, *H. annuus*, *Oryza sativa*, and *Zea mays* were obtained from the NCBI database to construct a maximum likelihood phylogenetic tree using MEGA 11 software [37] with 500 bootstrap replicates. Multiple-sequence alignments of CYC1 proteins were performed with ClustalW 2.0 software [38] followed by image processing with GeneDoc 2.7 software [39].

### 2.4. Quantitative Real-Time PCR

Consistent with our previous study [33], the flowering process of chrysanthemum was dived into five stages. Ray and disc florets were taken from the capitulum at stage1, stage 3, and stage 5 for comparative analysis of gene expression. The involucral bract, receptacle, ray petal, disc petal, pistil, and stamen samples were taken from chrysanthemum separately at stage 4 and stage 5 for floral tissue-specific gene expression analysis. In addition, the stamen samples were collected from disc florets, and the pistil samples (including ovary, style, and stigma) were pooled from ray and disc florets. cDNA was synthesized using a RT reagent Kit with gDNA Eraser (Perfect Real Time; TaKaRa, Shiga, Japan) after the total RNA was extracted and measured. Quantitative real-time PCR (qPCR) was carried out as described before with three biological replicates [40]. The primers for qPCR (*CmCYC1a*-F2/R2) are listed in Table S1. The transcript level of *P2Acs* [41] was used as a quantitative control and the relative expression level of *CmCYC1a* was calculated with the 2^–ΔΔCt^ method [42]. The significant differences were determined by using SPSS 20.0 software.

### 2.5. Arabidopsis Transformation

The CDS of *CmCYC1a* was amplified with *CmCYC1a*-F3/R3 (Table S1) and ligated into a pCambia1304 vector [33], introduced into the *Agrobacteriaum umefaciens* strain GV3101, and then transformed into *A. thaliana* [43]. The transgenic plants were selected using MS medium with hygromycin B (50 mg/L), and the homozygous T3 lines were validated by PCR with CmCYC1a-F1/R1 and qPCR with *CmCYC1a*-F2/R2. The reference gene used here was *AtActin*. Thirty flowers of each line were randomly selected for observation and stamen counting. The significant differences were determined by using SPSS 20.0 software.

### 2.6. Subcellular Localization

The CDS of *CmCYC1a* was amplified with *CmCYC1a*-F4/R4 (Table S1) and ligated into a pSuper1300-GFP vector, introduced into the *A. umefaciens* strain GV3101, and injected into tobacco leaves [44]. The GFP fluorescence was visualized using a confocal microscope (Leica, Wetzlar, Germany).

### 2.7. Y2H Assay

With the primers listed in Table S1, the CDSs of *CmCYC1a* and *CmCYC2* were amplified and cloned into pGBKT7 and pGADT7 vectors. Y2H assays were conducted as described before [40]. Y2H screenings of each protein combination were performed three times.

## 3. Results

### 3.1. Identification and Analysis of CmCYC1a

**Figure 1:**
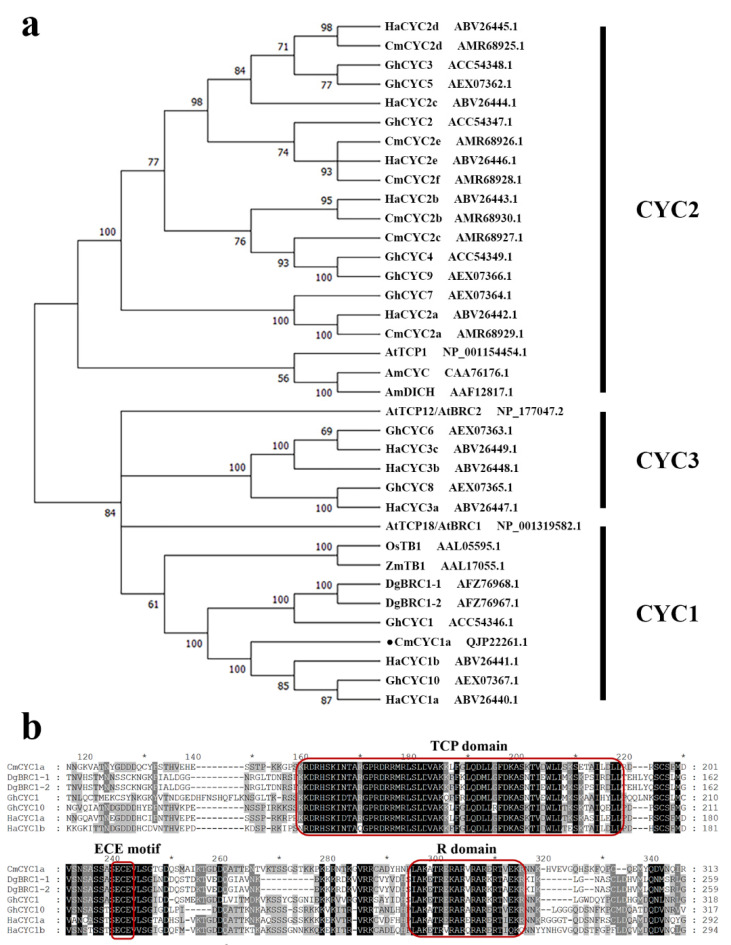
Phylogenetic analysis and sequence alignment of CmCYC1a. (**a**) Maximum likelihood tree of CmCYC1a and other ECE proteins from *Antirrhinum majus*, *Arabidopsis thaliana*, *Chrysanthemum morifolium*, *Gerbera hybrida*, *Helianthus annuus*, *Oryza sativa*, and *Zea mays*. (**b**) Multiple alignment of CmCYC1a and CYC1-like sequences from *C. morifolium*, *G. hybrida*, and *H. annuus*. TCP domain, ECE motif, and R domain are boxed in red.

The CDS of *CmCYC1a* (1095 bp) was isolated from the inflorescences of the chrysanthemum cultivar ‘Fen Ditan’. Based on the cDNA sequences, *CmCYC1a* is annotated to encode a 364 amino acid protein. Phylogenetic analysis of CmCYC1a and other ECE members (Figure 1a) confirmed that CmCYC1a belongs to the CYC1 subclade and is closely related to GhCYC10 (*G. hybrida*), HaCYC1a, and HaCYC1b (*H. annuus*). In addition, multiple-sequence alignment of CmCYC1a with other CYC1 members (Figure 1b) revealed that CmCYC1a harbors a typical TCP domain and an arginine-rich R domain [45], with a complete ECE (glutamic acid–cysteine–glutamic acid) motif [4] between them.

### 3.2. Expression Analysis of CmCYC1a in C. morifolium

**Figure 2:**
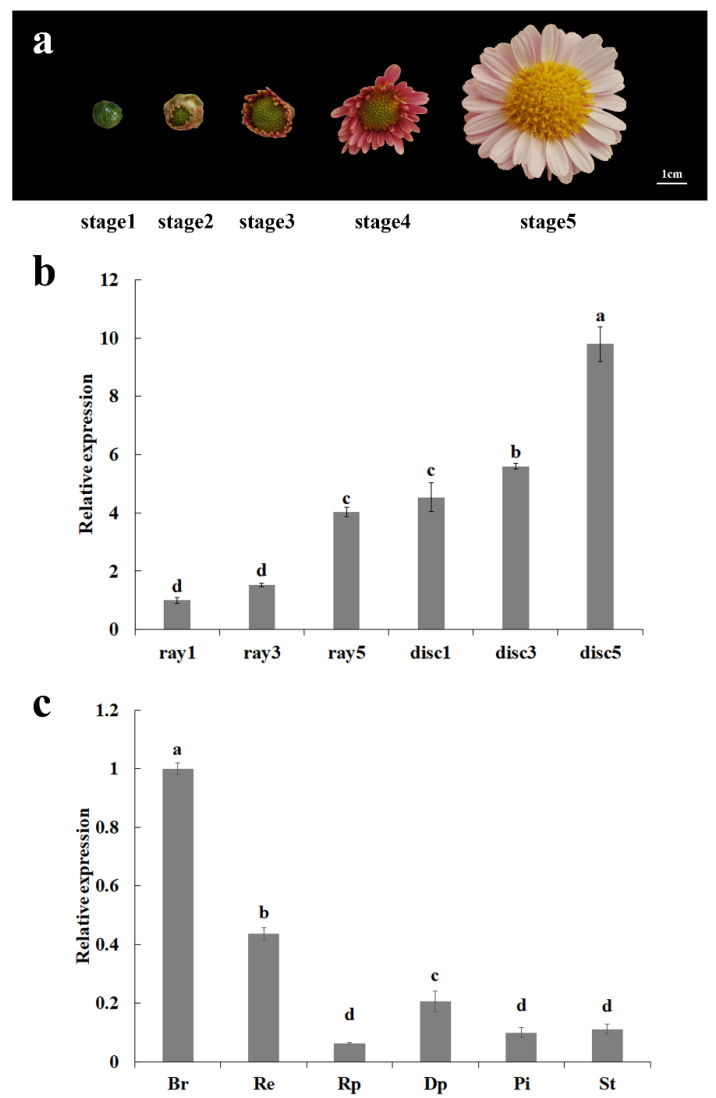
Expression analysis of *CmCYC1a* in *C. morifolium* ‘Fen Ditan’ during flowering process. (**a**) Capitulum morphology of ‘Fen Ditan’ at five stages of flowering; (**b**) relative expression level of *CmCYC1a* in ray and disc florets at stage 1, stage 3, and stage 5; (**c**) relative expression level of *CmCYC1a* in different tissues. *Br* involucral bract, *Re* receptacle, *Rp* ray petal, *Dp* disc petal, *Pi* pistil, and *St* stamen. Error bar: standard deviation; different lowercase letters: significant differences (Duncan’s test, *p* < 0.05).

To investigate the expression pattern of *CmCYC1a*, qPCR analysis was performed. Overall, compared with ray florets, *CmCYC1a* was expressed at higher levels in disc florets. In both disc and ray florets, the expression of *CmCYC1a* was gradually increased with the flowering process, reaching the highest expression level at stage 5 (Figure 2a,b). To explore the expression of *CmCYC1a* in a broader context, the *CmCYC1a* transcript levels were also detected in floral organs of chrysanthemum at later stages of flowering (Figure 2c). Basically, the expression of *CmCYC1a* was detected in all tissues, with higher expression in involucral bracts and receptacles. Furthermore, the expression level of *CmCYC1a* in disc petals was higher than in ray petals.

### 3.3. Ectopic Expression of CmCYC1a in A. thaliana Altered Flower Symmetry and Stamen Number

**Figure 3:**
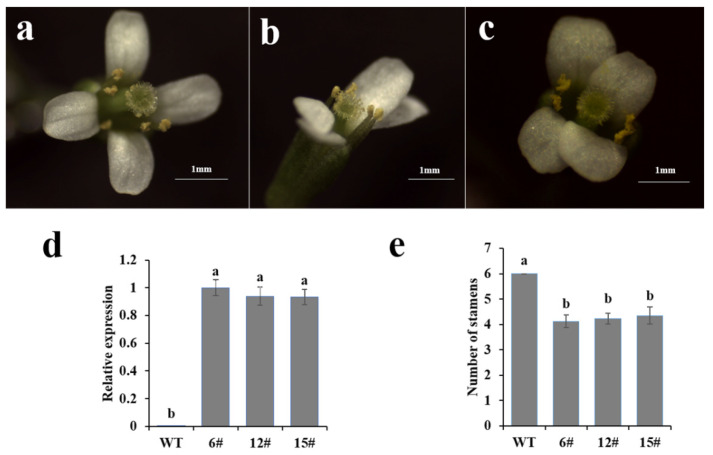
Ectopic expression of *CmCYC1a* in *Arabidopsis*. (**a**) *Arabidopsis* flower (wild type, WT); (**b**,**c**) flower phenotypes in transgenic lines; (**d**) qPCR results of *CmCYC1a* expression in flowers of WT and three transgenic lines; (**e**) number of stamens in WT and transgenic lines. Error bar: standard deviation; different lowercase letters: significant differences (Duncan’s test, *p* < 0.05).

To explore the function of *CmCYC1a* in floral development, *CmCYC1a* was overexpressed in *Arabidopsis* (Columbia). Three *35S:: CmCYC1a* lines (line 6, 12 and 15) were confirmed by PCR (Figure S2) and qPCR (Figure 3d) and selected for detailed analysis. Ectopic expression of *CmCYC1a* altered *Arabidopsis* flower symmetry from polysymmetric (Figure 3a) to monosymmetric (Figure 3b,c). In addition, the stamen number was reduced from six to four or five (Figure 3e). These results indicate the function of *CmCYC1a* in regulating flower symmetry and stamen development.

### 3.4. Protein–Protein Interactions Between CmCYC1a and CmCYC2 Transcription Factors

**Figure 4:**
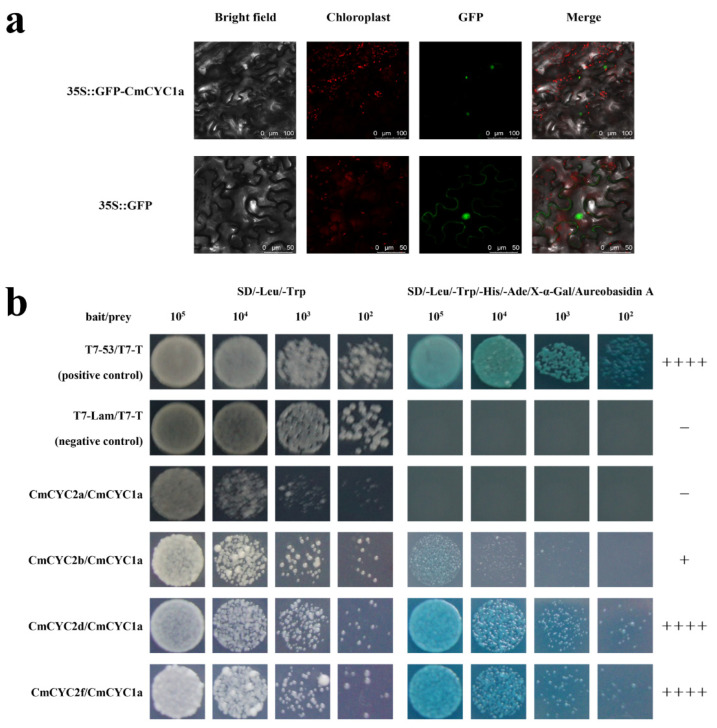
Interactions between CmCYC1a and CmCYC2. (**a**) Subcellular localization of CmCYC1a; (**b**) Y2H assays show three pairs of protein–protein interaction: CmCYC1a-CmCYC2b, CmCYC1a-CmCYC2d, and CmCYC1a-CmCYC2f. Intensity of interaction is denoted by “+”, and “−” means no interaction.

It is speculated that the regulatory functions of some TCP proteins may depend on interactions with other proteins [3]. Given that *CmCYC1a* and *CmCYC2* have some overlapping expression patterns during flower development [33] and somewhat similar phenotypes after overexpression in *Arabidopsis* [29], we examined protein–protein interactions between CmCYC1a and CmCYC2. GFP fluorescence in Figure 4a shows that CmCYC1a was mainly localized in the nucleus, consistent with CmCYC2 [33]. In Y2H assays, CmCYC1a could autonomously activate the reporter genes, like CmCYC2c and CmCYC2e, making them unable to be baits. Y2H interaction studies revealed that CmCYC1a can strongly dimerize with CmCYC2d and CmCYC2f, while the interaction between CmCYC1a and CmCYC2b is weak (Figure 4b). Taking all the results together, we propose three pairs of protein–protein interaction, which are CmCYC1a-CmCYC2b, CmCYC1a-CmCYC2d, and CmCYC1a-CmCYC2f.

## 4. Discussion

Prior studies of the *CYC1*-like ortholog *ZmTB1* in maize found that *ZmTB1* controls apical dominance, arrests stamen growth, and generates female inflorescences [16]. The function of *CYC1*-like orthologs in preventing branch outgrowth has been validated in many other species, including both monocots and dicots, such as *O. sativa* (*OsTB1*) [46], *A. thaliana* (*AtBRC1/AtTCP18*) [18], *Solanum lycopersicum* (*SlBRC1*) [47], *C*. *morifolium* (*DgBRC1*) [30], and *Medicago truncatula* (*MtTCP18*) [48]. However, few studies have reported the role of CYC1 genes in floral development. In Papaveraceae, two *PAPACYL* paralogs, closest to *AtBRC1/AtTCP18*, are expressed during flower development in three species that differ in flower symmetry. Interestingly, *PAPACYL* transcripts are asymmetrically expressed in the outer petals of the monosymmetric species and expressed in the developing anther of the bisymmetric species, suggesting a correlation with flower symmetry and stamen development [49]. In *K. macedonica*, *KmCYC1* is expressed significantly more in the lateral and dorsal petals compared with the ventral petals of external florets, indicating a possible association with flower symmetry [27]. Transcriptome analysis of the chrysanthemum cultivar ‘Jinba’ has identified two CYC1 genes and they were both highly expressed in immature inflorescences, suggesting their involvement in inflorescence formation [25].

In this study, a CYC1 gene *CmCYC1a* was isolated in the chrysanthemum cultivar ‘Fen Ditan’ and the expression level of *CmCYC1a* was increased successively during the flowering process in both disc and ray florets, suggesting that *CmCYC1a* is associated with the growth of the two florets or the chrysanthemum capitulum. Additionally, *CmCYC1a* was expressed at higher levels in disc florets than in ray florets, consistent with the sunflower *HaCYC1a*. Actually, in both gerbera and sunflower, *HaCYC1a* is the only CYC1 gene with differential expression between different flower types [11], indicating the involvement of *CmCYC1a* and *HaCYC1a* in the differentiation of flower types. Floral tissue-specific expression analysis at later stages of flowering found that *CmCYC1a* was expressed in all of the tissues examined, suggesting its extensive involvement in chrysanthemum flower growth and development.

To further explore the functions of *CmCYC1a*, the gene was overexpressed in *Arabidopsis*. In *35S::CmCYC1a* lines, the flowers were altered from radially symmetrical to bilaterally symmetrical with fewer stamens. In *Arabidopsis* with ectopic expression of three *Canna indica* ECE genes, a similar phenotype was reported. Moreover, the altered flower symmetry of transgenic *Arabidopsis* is thought to be caused by the loss of stamens [50]. On the other hand, our previous study found that overexpression of *CmCYC2* in the *tcp1* mutant also changed flower symmetry but not the number of stamens [29]. And overexpression of two transcripts of *Cyc2CL*, a CYC2 gene cloned from ‘Fen Ditan’, resulted in the inhibition of petal and stamen development in *Arabidopsis* [35]. Since the ancient paralogues may stay in the same regulatory network [51], our results indicate that *CmCYC1a* may have retained the *CYC1*-like gene function in suppressing stamen growth and is functionally redundant along with *CmCYC2* in regulating floral symmetry and stamen development. Indeed, it has been proposed that *TCP1* may act redundantly along with *BRC1/2* in leaf development [52]. The redundant functions of gerbera CYC2 genes *GhCYC2/3/4* have been demonstrated, and their ectopic expression in gerbera disc florets resulted in transition to ray-like florets and the inhibition of stamen development [12]. Therefore, our speculations can be further verified in chrysanthemum with CRISPR/Cas9 [53] or other plant transformation methods [54].

Protein–protein interactions showed a complex network involving CYC1 and CYC2 proteins in chrysanthemum. Our previous study has confirmed that CmCYC2b can form heterodimers with CmCYC2d and CmCYC2e, and CmCYC2c can form a heterodimer with CmCYC2d [29]. Here, CmCYC1a-CmCYC2b, CmCYC1a-CmCYC2d, and CmCYC1a-CmCYC2f were revealed in the Y2H assays, suggesting that CmCYC2b and CmCYC2d may be key factors in the network linking the two subclades, and CmCYC1a and CmCYC2 may participate together in capitulum morphogenesis through protein interactions. Notably, the interactions observed in the Y2H assays can be further validated by other experiments such as bimolecular fluorescence complementation (BiFC) [55] and surface plasmon resonance (SPR) [56]. In addition, overexpression of *CmWUS*, a *WUSHCHEL*-like gene, also affects flower symmetry and inhibits stamen and pistil development in *Arabidopsis,* and CmWUS can interact with CmCYC2d [33]. The Y2H assays performed in *C. lavandulifolium* have found that A-class MADs-box proteins DenFUL and ClAP2 can form heterodimers with ClCYC2d and ClCYC2e [57]. In gerbera, the C and E class protein pair GAGA1-GRCD5 may suppress the expression of *GhCYC3*, which specifies ray floret identity, and affect stamen development [58]. Furthermore, the negative feedback loop between *WUS* and *AGAMOUS (AG)*, a C class gene, has been established in *Arabidopsis* [59,60]. Hence, a more complex molecular regulatory network of floral development in chrysanthemum encompassing ECE, WUS, and MADS-box has emerged and is waiting to be explored.

In particular, *CmCYC1a* was highly expressed in involucral bracts and receptacles, as was the previously reported *Cyc2CL-1* in ‘Fen Ditan’ [35]. *CmCYC2* and Gerbera ECE genes, like *GhCYC2*, *GhCYC9,* and *GhCYC10*, are also highly expressed in involucral bracts [11]. In *C. lavandulifolium*, *ClSVPa*, a MIKCc-type MADS-box gene, is specifically expressed in involucral bracts and receptacles [61]. The protein–protein interaction between ClCYC2e and DenFUL is thought to have a possible role in receptacle identity [57]. In some T1 plants of *35S::CmCYC1a*, the flowers showed strong phenotypes (Figure S3) with disrupted floral organs, including abnormal sepals, similar with *Arabidopsis* overexpressing *Cyc2CL* [35] or *GhCYC4/7* [12] and *SVP* mutants in the CRISPR/Cas9 system [62], indicating other functions of *CmCYC1a* in floral development. But due to floral abortion, these T1 plants could not be screened and observed afterwards. Thus, the specific role of *CmCYC1a* in the development of involucral bracts and receptacles and its relationship with CYC2 and MADS-box genes are unclear and await more research for elucidation.

TCP transcription factors are mediators of hormone activity and key players in hormone signaling [63]. In the promoter region of *C. lavandulifolium* TCP genes, many hormone response elements were found [26]. The critical role of auxin concentration in determining floret identity has already been reported in the studies of *Senecio vulgaris* and *Matricaria inodora*. Additionally, the expression of *MiRAY2*, a CYC2 gene, is affected by auxin [64]. Integrated transcriptomics and metabolomics analyses also revealed a significant correlation between auxin and genes related to flower development, such as TCP and MADs-box genes, in *Argyranthemum frutescens* (Asteraceae) [65]. The expression of the CYC1 gene *BRC1* is influenced by diverse phytohormones, including auxin, cytokinins, strigolactones, and gibberellin, but the detailed mechanism is still confusing [66]. In chrysanthemum, the transcript level of *DgBRC1* is also associated with auxin and cytokinin [30,67]. We propose that *CmCYC1a* may be involved in hormonal signaling networks, especially in relation to auxin, during flower development, but this speculation needs to be further verified.

## 5. Conclusions

In the present study, an ECE-CYC1 gene, *CmCYC1a*, was isolated and characterized in *C. morifolium*. Compared with ray florets, *CmCYC1a* was expressed at higher levels in disc florets and the expression of *CmCYC1a* was increased in both florets during the flowering process, suggesting its association with the growth of chrysanthemum florets. Overexpression of *CmCYC1a* in *Arabidopsis* changed the flower symmetry from actinomorphic to zygomorphic and reduced the number of stamens, similar to the phenotypes of overexpression of *CmCYC2* genes. Additionally, Y2H assays revealed that CmCYC1a can interact with CmCYC2b, CmCYC2d, and CmCYC2f. The results provide evidence for the involvement of *CmCYC1a* in regulating flower symmetry and stamen development and interactions between CmCYC1a and CmCYC2 transcription factors. We hypothesize that CmCYC1a and CmCYC2 have partially redundant functions in floral development and participate together in capitulum morphogenesis through protein interactions. Our findings may help to clarify the complex molecular mechanism of chrysanthemum inflorescence, broaden our knowledge of the contributions of ECE genes in capitulum formation, and provide a molecular basis for the enrichment of chrysanthemum flower type.

## Data Availability

The original contributions presented in this study are included in the article/Supplementary Materials. Further inquiries can be directed to the corresponding authors.

## Supplementary Materials

The following supporting information can be downloaded at https://www.mdpi.com/article/10.3390/genes16020152/s1: Figure S1: Melt curves of *CmCYC1a* and *PP2Acs* in qPCR. Figure S2: Identification of three *35S:: CmCYC1a* lines via PCR. Figure S3: Strong phenotypes in *35S:: CmCYC1a* T1 generation. Table S1: Primers used.
