# Supplementary material for: ECE-CYC1 Transcription Factor CmCYC1a May Interact with CmCYC2 in Regulating Flower Symmetry and Stamen Development in Chrysanthemum morifolium
Source: Genes (Basel). 2025 Jan 26;16(2):152. doi: 10.3390/genes16020152 (PMC11855172; doi:10.3390/genes16020152)
Supplement: Supplementary file 1 [file genes-16-00152-s001.zip › genes-3437764-supplementary.pdf]

*Supplementary Material*

# ECE-CYC1 Transcription Factor CmCYC1a May Interact with CmCYC2 in Regulating Flower Symmetry and Stamen Development in *Chrysanthemum morifolium*

**Yi Yang [1,2,*], Ming Sun [2], Cunquan Yuan [2] and Qixiang Zhang [2,*]**

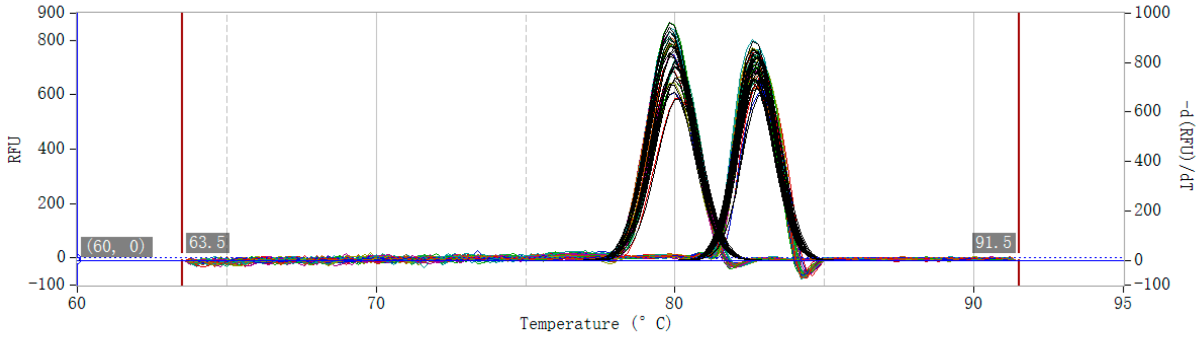


**Figure S1:** Melt curves of *CmCYC1a* and *PP2Acs* in qPCR. The amplicons are 132 bp and 171 bp, respectively.

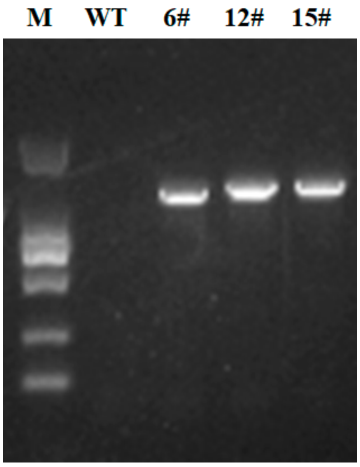


**Figure S2:** Identification of three *35S:: CmCYC1a* lines via PCR. M, DL2000 DNA marker.

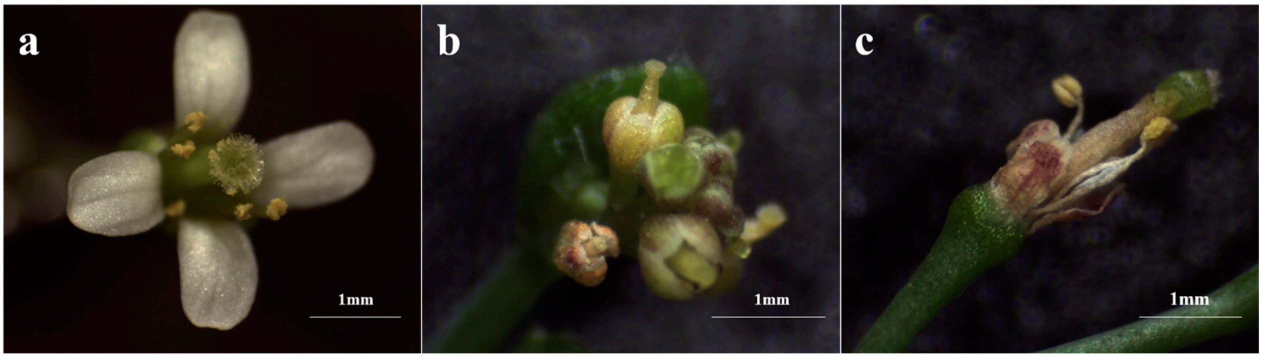


**Figure S3:** Strong phenotypes in *35S:: CmCYC1a* T1 generation. (**a**) *Arabidopsis* flower (WT); (**b, c**) flower phenotypes of T1 plants (transgenic line 6). The development of floral organ was disrupted and failed to produce normal seeds.

**Table S1. Primers used**

| Primer name | DNA sequence (5′-3′) | Experiment |
|---|---|---|
| *CmCYC1a*-F1 | ATGTATCCCTCTTCAAACAACAACC | *CmCYC1a* CDS cloning |
| *CmCYC1a*-R1 | CTAGTTATTGAACAAGTAATTTGTT | |
| *CmCYC1a*-F2 | CGGAACTGGTGATCAGTCTATG | qRCR |
| *CmCYC1a*-R2 | GCACACCGTCTAACTCCTTTA | |
| *PP2Acs*-F | ATCAGAACAGGAGGTCAGGG | |
| *PP2Acs*-R | TAATTTGTATCGGGGCACTT | |
| *CmCYC1a*-F3 | GGACTCTTGACCATGGTTATGTATCCCTCTTCAAACAACAACC | Overexpression of *CmCYC1a* in *Arabidopsis thaliana* |
| *CmCYC1a*-R3 | ATTCGAGCTGGTCACCCTAGTTATTGAACAAGTAATTTGTT | |
| *AtACTIN*-F | GGTATGGGTCAGAAAGATGCT | |
| *AtACTIN*-R | CGTTGTAGAAAGTGTGATGCC | |
| *CmCYC1a*-F4 | AGGGGCCCGGGGTCGACATGTATCCCTCTTCAAACAACAACC | Subcellular localization |
| *CmCYC1a*-R4 | GGTACCGGATCCACTAGTGTTATTGAACAAGTAATTTGTT | |
| *CmCYC1a*-F5 | CATGGAGGCCGAATTCATGTATCCCTCTTCAAACAACAACC | Y2H assay pGBKT7 construction |
| *CmCYC1a*-R5 | GCAGGTCGACGGATCCCTAGTTATTGAACAAGTAATTTGTT | |
| *CmCYC2a*-F | CATGGAGGCCGAATTCATGTTTAATTCTTCAAATCC | |
| *CmCYC2a*-R | GCAGGTCGACGGATCCTTAATCCTCTTTCGATTCAA | |
| *CmCYC2b*-F | CATGGAGGCCGAATTCATGTTTTCATCAAATCTTTTTCCA | |
| *CmCYC2b*-R | GCAGGTCGACGGATCCTTAAGTTCTTTGCTGTTCCA | |
| *CmCYC2c*-F | CATGGAGGCCGAATTCATGTTTTCCTCAAACCCCTT | |
| *CmCYC2c*-R | GCAGGTCGACGGATCCTTACAACATCAGTCCAGGTC | |
| *CmCYC2d*-F | CATGGAGGCCGAATTCATGTTTTCCTCGAACCCTT | |
| *CmCYC2d*-R | GCAGGTCGACGGATCCCTAGTGTAAATTTAGGAAACTTGTG | |
| *CmCYC2e*-F | CATGGAGGCCGAATTCATGTTTTCCACAAATCCCTATTCAC | |
| *CmCYC2e*-R | GCAGGTCGACGGATCCCTAAATAGGGTGACGGTCGC | |
| *CmCYC2f*-F | CATGGAGGCCGAATTCATGATGTTTTCCCCAAACCA | |
| *CmCYC2f*-R | GCAGGTCGACGGATCCCTACTTGTTTAAACAAAGACCTTGA | |
| *CmCYC1a*-F6 | GGAGGCCAGTGAATTCATGTATCCCTCTTCAAACAACAACC | Y2H assay pGADT7 construction |
| *CmCYC1a*-R6 | CGAGCTCGATGGATCCCTAGTTATTGAACAAGTAATTTGTT | |
